# Vanillic Acid, a Bioactive Phenolic Compound, Counteracts LPS-Induced Neurotoxicity by Regulating c-Jun N-Terminal Kinase in Mouse Brain

**DOI:** 10.3390/ijms22010361

**Published:** 2020-12-31

**Authors:** Rahat Ullah, Muhammad Ikram, Tae Ju Park, Riaz Ahmad, Kamran Saeed, Sayed Ibrar Alam, Inayat Ur Rehman, Amjad Khan, Ibrahim Khan, Min Gi Jo, Myeong Ok Kim

**Affiliations:** 1 Division of Life Sciences and Applied Life Science (BK 21plus), College of Natural Science, Gyeongsang National University, Jinju 52828, Korea; rahatullah1414@gnu.ac.kr (R.U.); qazafi417@gnu.ac.kr (M.I.); riazk0499@gnu.ac.kr (R.A.); kamran.biochem@gnu.ac.kr (K.S.); ibrar@gnu.ac.kr (S.I.A.); inayaturrehman201516@gnu.ac.kr (I.U.R.); amjadkhan@gnu.ac.kr (A.K.); ibrahim1994@gnu.ac.kr (I.K.); mingi.cho@gnu.ac.kr (M.G.J.); 2 Haemato-Oncology/Systems Medicine Group, Paul O’Gorman Leukaemia Research Centre, Institute of Cancer Sciences, College of Medical, Veterinary and Life Sciences (MVLS), University of Glasgow, Glasgow G12OZD, UK; 2358860P@student.gla.ac.uk

**Keywords:** vanillic acid, lipopolysaccharide, c-Jun N-terminal kinases, neuroinflammation, amyloidogenesis, synaptic and memory impairment, neurodegenerative diseases

## Abstract

The receptor for advanced glycation end products (RAGE), a pattern recognition receptor signaling event, has been associated with several human illnesses, including neurodegenerative diseases, particularly in Alzheimer’s disease (AD). Vanillic acid (V.A), a flavoring agent, is a benzoic acid derivative having a broad range of biological activities, including antioxidant, anti-inflammatory, and neuroprotective effects. However, the underlying molecular mechanisms of V.A in exerting neuroprotection are not well investigated. The present study aims to explore the neuroprotective effects of V.A against lipopolysaccharides (LPS)-induced neuroinflammation, amyloidogenesis, synaptic/memory dysfunction, and neurodegeneration in mice brain. Behavioral tests and biochemical and immunofluorescence assays were applied. Our results indicated increased expression of RAGE and its downstream phospho-c-Jun n-terminal kinase (p-JNK) in the LPS-alone treated group, which was significantly reduced in the V.A + LPS co-treated group. We also found that systemic administration of LPS-injection induced glial cells (microglia and astrocytes) activation and significantly increased expression level of nuclear factor kappa-light-chain-enhancer of activated B cells (NF-KB) and secretion of proinflammatory cytokines including tumor necrosis factor alpha (TNF-α), interleukin-1 β (IL1-β), and cyclooxygenase (COX-2). However, V.A + LPS co-treatment significantly inhibited the LPS-induced activation of glial cells and neuroinflammatory mediators. Moreover, we also noted that V.A treatment significantly attenuated LPS-induced increases in the expression of AD markers, such as β-site amyloid precursor protein (APP)–cleaving enzyme 1 (BACE1) and amyloid-β (Aβ). Furthermore, V.A treatment significantly reversed LPS-induced synaptic loss via enhancing the expression level of pre- and post-synaptic markers (PSD-95 and SYP), and improved memory performance in LPS-alone treated group. Taken together; we suggest that neuroprotective effects of V.A against LPS-induced neurotoxicity might be via inhibition of LPS/RAGE mediated JNK signaling pathway; and encourage future studies that V.A would be a potential neuroprotective and neurotherapeutic candidate in various neurological disorders.

## 1. Introduction

Inflammation is the body’s defensive immune response to harmful stimuli and is classified into acute and chronic types. The acute form is an immediate immune response to central nervous system (CNS) insults, whereas the chronic form is a prolonged immune response associated with damage to normal tissues [1]. Both the immune system components (innate and adaptive immunity) play a crucial role in the development of neuroinflammation [2,3]. There is substantial debate describing the role of neuroinflammation in the pathology of many neurological diseases such as Alzheimer’s disease (AD), amyotrophic lateral sclerosis, and Parkinson’s disease [4]. Within the CNS, neuroinflammation mostly involved two forms of brain’s local (resident) innate immune cells (glial cells; microglia and astrocytes) [5]. Tissue damage and systemic inflammation results in activation of these glial cells [6] that intern triggers several inflammatory cytokines, which lead to neuroinflammatory neurodegeneration [7].

The receptor for advanced glycation endproducts (RAGE), a member of the supergene family of immunoglobins, is a transmembrane receptor expressed on various cell types within the brain, including glial cells [8,9]. It is a multiligand receptor that binds/interacts with a wide range of ligands, including lipopolysaccharides (LPS; an endotoxin/a major component of the gram-negative cell wall and a potent inducer of inflammation) [10,11,12]. Both in-vivo and in-vitro studies have shown that LPS is a well-known and potent brain macrophage stimulator, which stimulates the immune system [13] that evokes inflammatory processes in the body and has detrimental effects on many vital organs, including the brain [14]. RAGE ligation activates a range of intracellular signaling pathways, including the activation of mitogen-activated protein kinases (MAPKs)/c-Jun N-terminal kinase (JNK), or nuclear factor kappa-light-chain-enhancer of activated B cells (NF-κB) pathway in initiation of the inflammatory process [15,16,17,18].

Importantly, in this regard, many studies have highlighted the significant role JNK pathway in preclinical AD models, including transgenic mice and AD patient’s brains [19,20]. Although JNK regulate various important brain functions including memory formation, repair, neuroinflammation and neuronal death [21]. However, abnormal activation of the JNK/c-Jun pathway ties with neuropathological features of AD. For instance, such signal transduction pathways regulate amyloid-β protein precursor (AβPP) phosphorylation (results in extracellular neuritic plaques formation from β-amyloid (Aβ) as well as mediate tau phosphorylation (results in intracellular neurofibrillary tangles formation from hyperphosphorylated tau protein) [19]. Likewise, the role of JNK pathway in Aβ and tau formation, other studies have also shown its role in synaptopathy and cognitive impairment [22,23], and its inhibition might reverse multiple pathological features and cognitive deficits associated with AD [20].

Natural sources have become the major target of researchers to search for potential effective therapeutic agents to combat inflammatory and neurological disorders diseases [24]. In this respect, considerable attention has been paid to plant phenolics [25]. Polyphenolics showed a wide variety of biological effects, including anti-microbial, anti-inflammatory, anti-cancer, anti-thrombotics, and neuroprotection [26]. Vanillin acid (V.A) (4-hydroxy-3-methoxybenzoic acid) are amongst the common phenolics well studied for their medicinal properties [25]. It is a benzoic acid derivative and is found in edible plants and fruits [27,28]. It is an oxidized form of vanillin and is also an intermediate in the production of vanillin from ferulic acid [29,30]. Numerous studies have shown that V.A has diverse pharmacological activities, including antihypertensive, antioxidant, and anti-inflammatory [25,28,31] anti-filarial, antimicrobial, and inhibition of snake venom activity [32].

Our laboratory previously investigated the antioxidant nature and possible mechanisms of V.A against amyloid-βeta (Aβ_1–42_)-induced oxidative stress-mediated cognitive deficits in mice. In this current preclinical study, the possible neuroprotective mechanism of V.A against LPS-induced glial cell activation, neuroinflammation, amyloidogenesis, and synaptic/memory impairments in different mice brain regions (cortex and hippocampus) were evaluated.

## 2. Results

### 2.1. Neuroprotective Effect of Vanillic Acid on Expression of LPS-Elevated RAGE Protein and Activated Gliosis in Mice Brains

**Figure 1:**
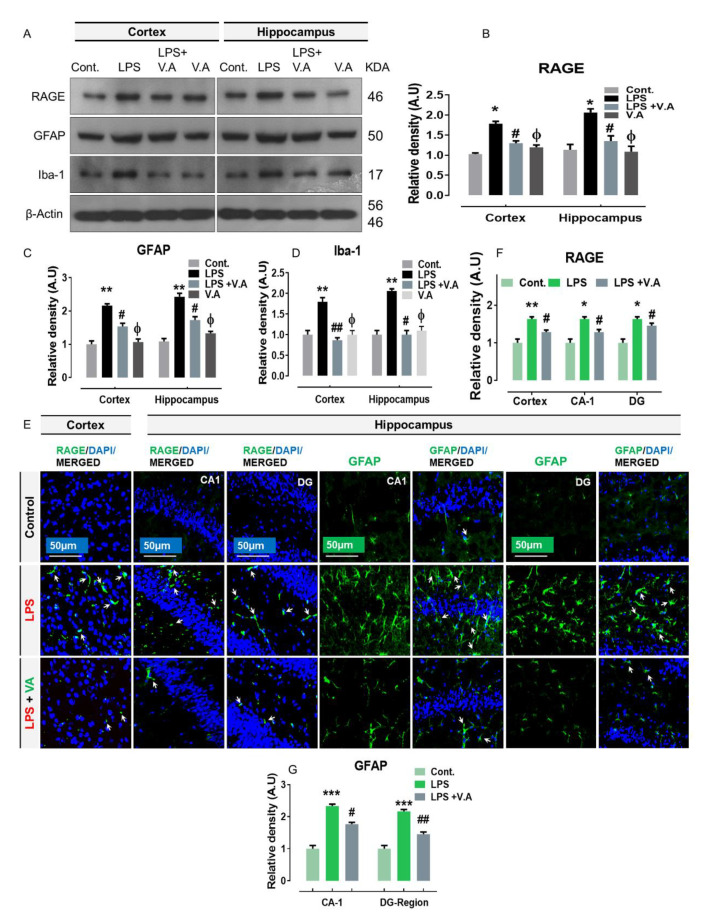
Vanillic acid treatment inhibited lipopolysaccharides (LPS)-activated receptor for advanced glycation endproducts (RAGE)/gliosis (microglia and astrocytes) in Mice Brain. (**A**–**D**) the western blot bands of the RAGE, glial fibrillary acidic protein (GFAP), and ionized calcium-binding adaptor molecule 1 (Iba-1) antibodies along with their relative histograms indicating their protein expression level in the cortex and hippocampus of mice brain. The western blot bands were cropped and quantified using ImageJ software, and the differences are shown in the histogram. The relative density values were expressed in comparison with control in arbitrary units (A.U). All the values were taken as the mean ± SEM of three repeated/independent experiments for the respective indicated protein. As a loading control, an anti-β-actin antibody was used. *n* = 8 mice/group, and the number (N) of experiments performed = 3. (**E**–**G**) the representative photomicrographs of immunofluorescence staining in different regions represent the immunoreactivity of RAGE (cortex, CA1, and dentate gyrus (DG) region) and GFAP (CA1 and DG) along with its relative histograms in various experimental brain groups (green, fluorescein isothiocyanate (FITC); blue, 4′, 6′-diamidino-2-phenylindole (DAPI)). White small arrows indicated the desired signals of the tested antibody. The relative integrated density values were expressed in comparison with control in A.U. All values were taken as the means (±S.E.M) for the respective indicated proteins. For nucleus staining, DAPI (blue) was used. *n* = 8 mice/group, and the number (N) of experiments = *N* = 3. Magnification = 10×. Scale bar; cortices/DG hippocampal regions = 50 μm. Asterisk sign (*) indicated significant difference from the normal saline-treated (Cont.) group; hash sign (#) indicated significant difference from LPS-alone treated group; while the phi sign (Φ) indicated no significance from normal saline-treated (Cont.) group. Significance: * # = *p* ≤ 0.05, ** ## = *p* ≤ 0.01, and *** = *p* ≤ 0.001.

RAGE, an important receptor for LPS (ligand), is expressed on almost all brain cells, including neurons, microglia, and astrocytes [12,33]. Many previous studies have shown that LPS (ligand/RAGE interactions) increases the expression level of RAGE protein [15,34] and therefore, stimulates glial cells (RAGE; expressed on microglia and astrocytes) in neuroinflammatory neurodegeneration [6]. Therefore, to analyze the effect of V.A on RAGE and gliosis (glial fibrillary acidic protein (GFAP), an astrocytes-marker, and ionized calcium-binding adaptor molecule 1 (Iba-1), a microglia marker), we carried out western blot and immunofluorescence analysis in all experimental groups. Our immunoblot results showed that compared to saline-treatment, systemic administration of LPS-injection significantly increased protein expression level of RAGE and number of activated astrocytes (GFAP; reactive cells), and microglia (Iba-1; reactive cells) in both cortex and hippocampus regions in LPS-alone treated group. Interestingly, V.A + LPS co-treatment significantly reduced the elevated expression of RAGE, GFAP, and Iba-1in comparison with LPS-alone treated group (Figure 1A–D). Additionally, immunoblot findings indicated that V.A was non-toxic to normal mice brain as no significant difference was found between normal saline-treated (control) mice and V.A alone-treated mice (Figure 1A). To further ascertain these results, we used confocal microscopy. In accordance with our immunoblot results, our immunofluorescence analysis also suggested that immunoreactivity of RAGE (cortex, CA1 (Cornu Ammonis), and dentate gyrus (DG)) and GFAP (CA1 and DG) was significantly increased in LPS-alone treated group in comparisons with normal saline-treated (control) mice. Interestingly, V.A + LPS co-treatment significantly reduced immunofluorescence reactivity of RAGE and activated GFAP in comparison with LPS-alone treated group (Figure 1E–G). Taken together, these results indicated that V.A is effective in preventing LPS-mediated higher expression of RAGE and activated gliosis in the mouse brain.

### 2.2. Neuroprotective Effect of Vanillic Acid on Expression of LPS-Activated p-JNK Protein in Mice Brains

**Figure 2:**
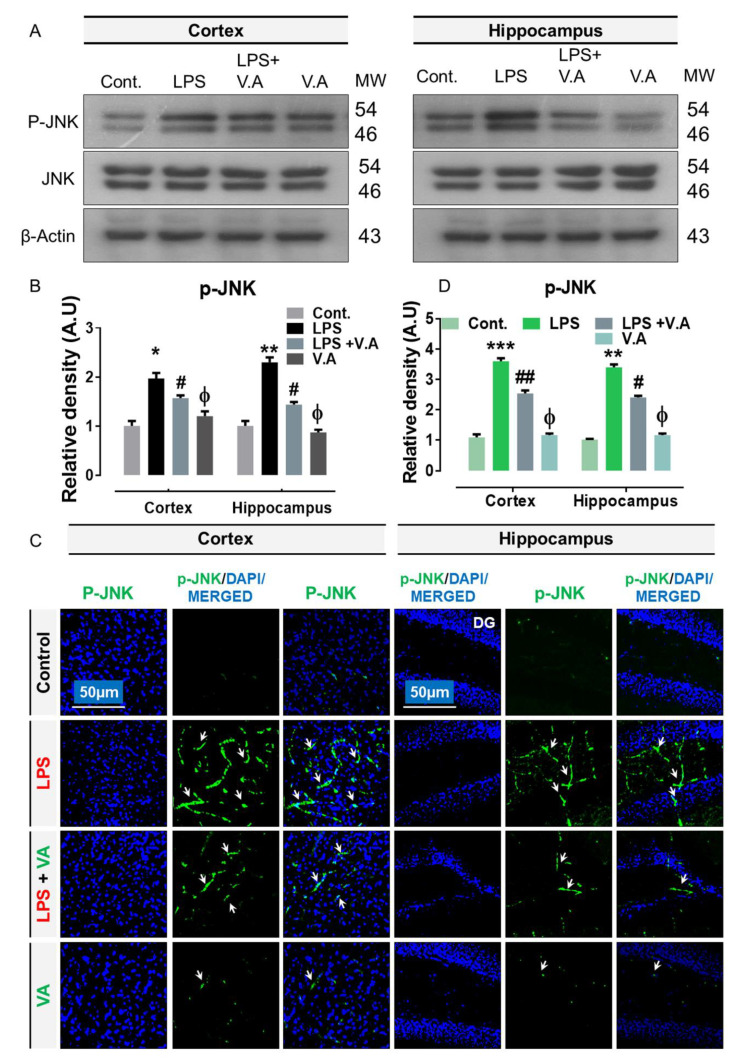
Vanillic acid treatment inhibited LPS-induced elevated expression level of stress kinase (c-Jun N terminal kinase (p-JNK)) in mice brain. (**A**,**B**) representative western blot band of p-JNK antibody along with its relative histogram indicating its protein expression level in both cortex and hippocampus regions of mice brain. The western blot bands were cropped and quantified using ImageJ software, and the differences are shown in the histogram. The relative density values were expressed in comparison with control in A.U. All values were taken as the mean ± SEM of three repeated/independent experiments for the respective indicated protein. As a loading control, an anti-β-actin antibody was used. *n* = 8 mice/group, and the number (*N*) of experiments performed (*N*) = 3. (**C**,**D**) the representative photomicrographs of immunofluorescence staining represent the immunoreactivity of p-JNK protein along with its relative histogram in different regions (cortex and hippocampus) in various experimental brain groups (green, FITC; blue, DAPI). White small arrows indicated the desired signals of the tested antibody. The relative integrated density values were expressed in comparison with control in A.U. All values were taken as the means (±S.E.M) for the respective indicated proteins. For nucleus staining, DAPI (blue) was used. *n* = 8 mice/group, and the number (*N*) of experiments = *N* = 3. Magnification = 10×. Scale bar; cortices/DG hippocampal regions = 50 μm. Asterisk sign (*) indicated significant difference from the normal saline-treated (Cont.) group; hash sign (#) indicated significant difference from LPS-alone treated group; while the phi sign (Φ) indicated no significance from normal saline-treated (Cont.) group. Significance: * # = *p* ≤ 0.05, ** ## = *p* ≤ 0.01, and *** = *p* ≤ 0.001.

Previously, studies have shown that ligand/RAGE interactions activate a range of intracellular signaling pathways, including the activation of MAPKs/JNK [17,35]. In addition, many studies have reported that LPS treatment increases the expression level of the stress associated kinases; importantly, c-Jun N terminal kinase (p-JNK) [24,36]. Therefore, in this study, to analyze the effect of V.A on LPS-activated p-JNK, we carried out western blot and immunofluorescence analysis in the experimental groups. Our immunoblot results showed that compared to saline-treatment, systemic administration of LPS-injection significantly increased protein expression level of p-JNK in both cortex and hippocampus regions in LPS-alone treated group. Interestingly, V.A + LPS co-treatment significantly reduced the elevated expression of p-JNK in comparison with LPS-alone treated group (Figure 2A,B).To further ascertain these results, we performed confocal microscopy. In accordance with our immunoblot results, our immunofluorescence analysis also suggested that p-JNK immunoreactivity was significantly increased (cortex, DG region of hippocampus) in LPS-alone treated group in comparisons with normal saline-treated (control) mice. Interestingly, V.A+ LPS co-treatment significantly reduced immunofluorescence reactivity of JNK in both the indicated regions in comparison with LPS-alone treated group (Figure 2C,D). Notably, both western blot and confocal analysis indicated that V.A was non-toxic as no significance difference was found between normal saline-treated (control) mice and V.A alone-treated mice (Figure 2). Taken together, these results indicated that V.A is effective and limit LPS-activated higher expression of p-JNK in the mouse brain.

### 2.3. Neuroprotective Effect of Vanillic Acid on LPS-Mediated p-NF-κB Activation and Associated Neuroinflammatory Markers/Cytokines in Mice Brains

**Figure 3:**
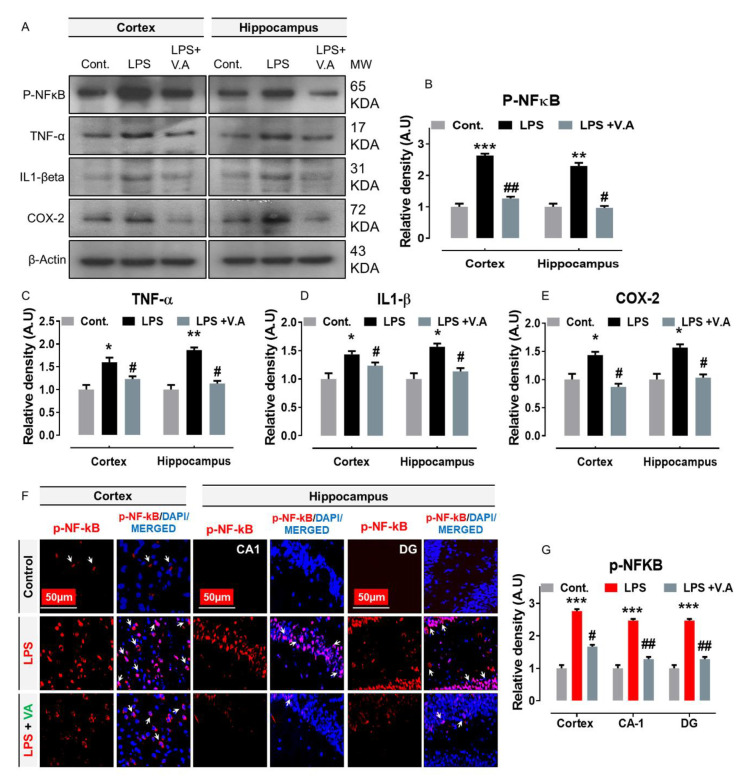
Vanillic acid treatment inhibited LPS-induced activation of nuclear factor nuclear factor-kappa B (p-NF-κB) and neuroinflammatory mediators/cytokines in mice brain. (**A**–**E**) the western blot bands of p-NF-κB, tumor necrosis factor alpha (TNF-α), interleukin-1 βeta (IL-1βeta), and cyclooxygenase-2 (COX-2) antibodies along with their relative histograms indicating their respective proteins expression level in both cortex and hippocampus regions of mice brain. The western blot bands were cropped and quantified using ImageJ software, and the differences are shown in the histogram. The relative density values were expressed in comparison with control in A.U. All values were taken as the mean ± SEM of three repeated/independent experiments for the respective indicated protein. As a loading control, an anti-β-actin antibody was used. *n* = 8 mice/group, and the number (*N*) of experiments performed (*N*) = 3. (**F**,**G**) the representative photomicrographs of immunofluorescence staining represent the immunoreactivity of p-NF-κB protein along with its relative histogram in different regions (cortex, CA1, and DG region) in various experimental brain groups (green, FITC; blue, DAPI). White small arrows indicated the desired signals of the tested antibody. The relative integrated density values were expressed in comparison with control in A.U. All values were taken as the means (±S.E.M) for the respective indicated proteins. For nucleus staining, DAPI (blue) was used. *n* = 8 mice/group, and the number (*N*) of experiments = *N* = 3. Magnification = 10×. Scale bar; cortices/DG hippocampal regions = 50 μm. Asterisk sign (*) indicated significant difference from the normal saline-treated (Cont.) group; hash sign (#) indicated significant difference from LPS-alone treated group. Significance: * # = *p* ≤ 0.05, ** ## = *p* ≤ 0.01, and *** = *p* ≤ 0.001.

Previously, studies have shown that ligand/RAGE interactions and activated p-JNK protein is critically involved in the upregulation of nuclear factor-kappa B (p-NF-κB; a transcription factor) [16,17], resulting in the production of pro-inflammatory cytokines and causing inflammation [37]. Importantly, in this regard, a considerable number of studies have also shown that systemic administration of LPS-injection increases the protein expression level of p-NF-κB and its associated neuroinflammatory markers in the initiation of neuroinflammation [7,36]. In order to analyze the effects of V.A on the LPS/JNK mediated NF-κB activation and its associated neuroinflammatory markers, we performed out western blotting and immunofluorescence analysis. Our immunoblot results showed that compared to saline-treatment, systemic administration of LPS-injection significantly increased protein expression level of p-NF-kB and its associated downstream neuroinflammatory mediators, including tumor necrosis factor alpha (TNFα), interleukin 1 beta (IL-1β), and cyclooxygenase-2 (COX-2) in cortex and hippocampus regions in LPS-alone treated group. Interestingly, V.A + LPS co-treatment significantly reduced the elevated expression of p-NF-kB and its associated downstream inflammatory mediators in comparison with LPS-alone treated group (Figure 3A–E). To further ascertain these results, we used confocal microscopy. In accordance with our immunoblot results, our immunofluorescence analysis also suggested that p-NF-kB immunoreactivity significantly increased (cortex, CA1, and DG region of hippocampus) in LPS-alone treated group in comparisons with normal saline-treated (control) mice. Interestingly, V.A + LPS co-treatment significantly reduced immunofluorescence reactivity of p-NF-kB in both the indicated regions in comparison with the LPS-alone treated group (Figure 3F,G). Taken together, these results indicated that V.A is effective against LPS-induced neuroinflammation might via inhibition of JNK-mediated p-NF-KB signaling pathway in the mouse brain.

### 2.4. Neuroprotective Effect of Vanillic Acid on Expression of LPS-Elevated BACE-1 and Aβ Proteins in Mice Brains

**Figure 4:**
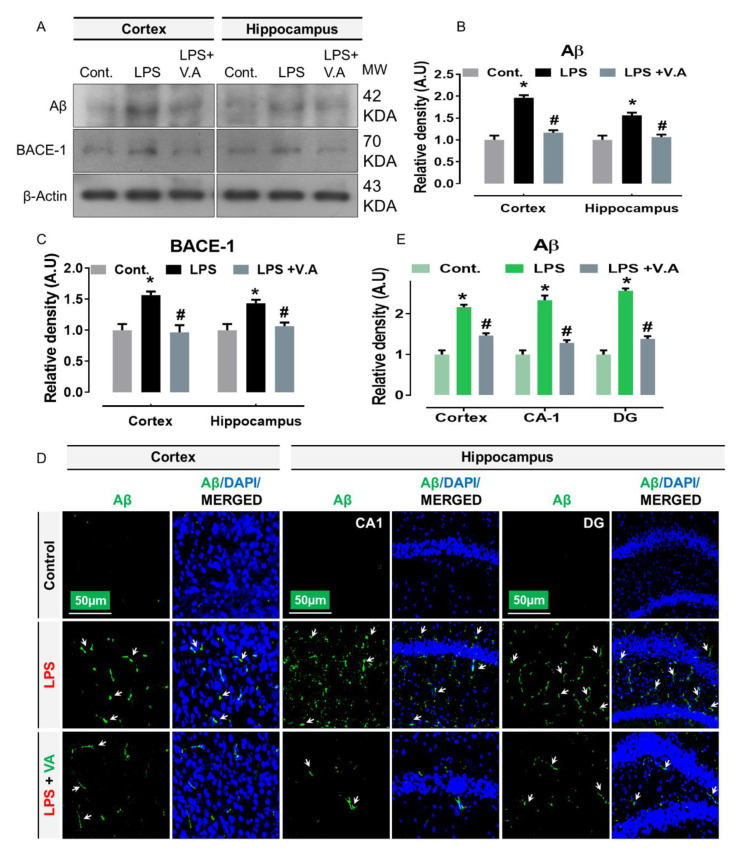
Vanillic acid treatment inhibits LPS-induced elevated protein expression of BACE-1 and β-amyloid (Aβ) in mice brain. (**A**–**C**) the western blot bands of BACE-1 and Aβ antibodies along with their relative histograms indicating their protein expression level in the cortex and hippocampus of mice brain. The western blot bands were cropped and quantified using ImageJ software, and the differences are shown in the histogram. The relative density values were expressed in comparison with control A.U. All values were taken as the mean ± SEM of three repeated/independent experiments for the respective indicated protein. As a loading control, an anti-β-actin antibody was used. *n* = 8 mice/group, and the number (*N*) of experiments performed = 3. (**D**,**E**) the representative photomicrographs of immunofluorescence staining represent the immunoreactivity of Aβ protein along with its relative histogram in different regions (cortex, CA1, and DG region) in various experimental brain groups (green, FITC; blue, DAPI). White small arrows indicated the desired signals of the tested antibody. The relative integrated density values were expressed in comparison with control in A.U. All values were taken as the means (±S.E.M) for the respective indicated proteins. For nucleus staining, DAPI (blue) was used. *n* = 8 mice/group, and the number (*N*) of experiments = *N* = 3. Magnification = 10×. Scale bar; cortices/DG hippocampal regions = 50 μm. Asterisk sign (*) indicated significant difference from the normal saline-treated (Cont.) group; hash sign (#) indicated significant difference from LPS-alone treated group. Significance: * # = *p* ≤ 0.05.

Early studies have indicated that the aberrant activation of JNK potentially contributes to the development of Aβ production, associated with AD [38]. Importantly, other reports have shown that systematic administration of LPS-injection has AD-like effects in rodents [14,39]. In order to analyze the effects of V.A on the LPS/JNK-mediated AD pathology, we performed western blotting and immunofluorescence analysis. Our immunoblot results showed that compared to saline-treatment, systemic administration of LPS-injection to wild type mice significantly increased protein expression level of beta-secretase 1 (BACE1), and Aβ in cortex and hippocampus regions in LPS-alone treated group. Interestingly, V.A + LPS co-treatment significantly reduced the elevated expression of BACE1 and Aβ in comparison with LPS-alone treated group (Figure 4A–C). To further ascertain these results, we used confocal microscopy. In accordance with our immunoblot results, our immunofluorescence analysis also suggested that Aβ immunoreactivity was significantly increased (cortex, CA1, and DG region of hippocampus) in LPS-alone treated group in comparisons with normal saline-treated (control) mice. Interestingly, V.A + LPS co-treatment significantly reduced immunofluorescence reactivity of Aβ in both the indicated regions in comparison with LPS-alone treated group (Figure 4D,E). Taken together, these results demonstrated that V.A is effective and exhibited a potent anti-amyloidogenic effect against LPS-induced AD pathology might occur via inhibition of JNK-signaling pathway in the mouse brain.

### 2.5. Neuroprotective Effect of Vanillic Acid on LPS-Induced Synaptic Dysfunction and Memory Impairment in Mice Brains

**Figure 5:**
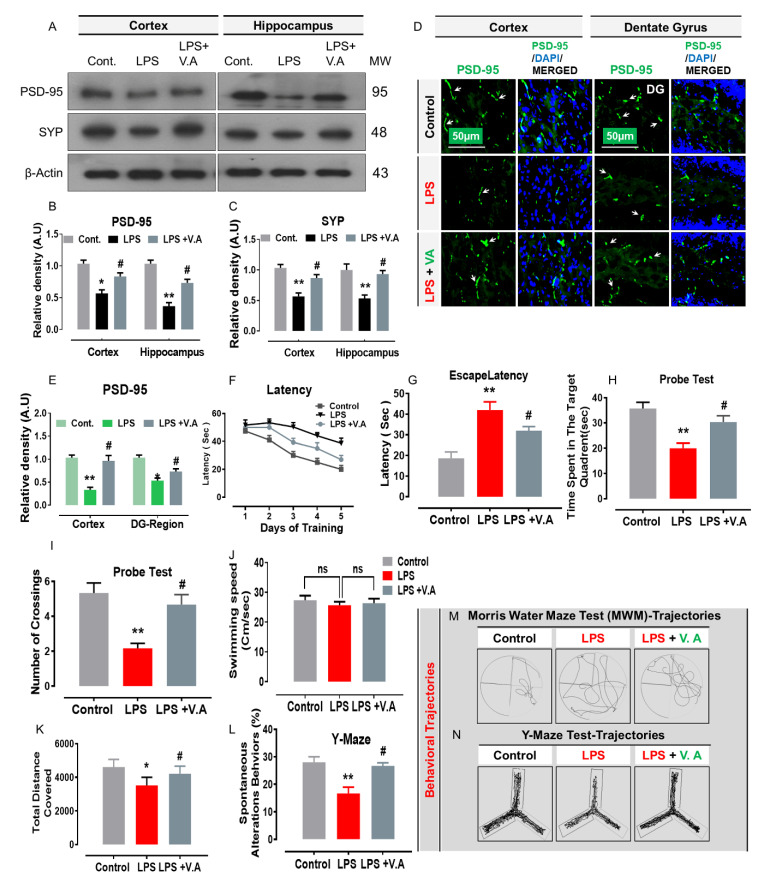
Vanillic acid treatment inhibited LPS-induced synaptic and memory dysfunction in mice brain. (**A**–**C**) the western blot bands of postsynaptic density proteins (PSD95), synaptophysin (SYP), antibodies along with their relative histograms indicating respective protein expression level in the cortex and hippocampus of mice brain. The western blot bands were cropped and quantified using ImageJ software, and the differences are shown in the histogram. The relative density values are expressed in comparison with control in A.U. All the values were taken as the mean ± SEM of three repeated/independent experiments for the respective indicated protein. As a loading control, an anti-β-actin antibody was used. *n* = 8 mice/group, and the number (*N*) of experiments performed = 3. (**D**,**E**) the representative photomicrographs of immunofluorescence staining represent the immunoreactivity of PSD95 in different regions (cortex and DG region) in various experimental brain groups (green, FITC; blue, DAPI). White small arrows indicated the desired signals of the tested antibody. The relative integrated density values were expressed in comparison with control arbitrary units (A.U). All values were taken as the means (±S.E.M) for the respective indicated proteins. For nucleus staining, DAPI (blue) was used. *n* = 8 mice/group, and the number (*N*) of experiments = *N* = 3. Magnification = 10×. Scale bar; cortices/DG hippocampal regions = 50 μm. Morris water maze (MWM) parameters are indicating (**F**,**G**) average escape latency (in a sec) and (**H**) probe test indicating time spent in target quadrant, (**I**) number of target crossings, and (**J**) Swim speed (cm/s) among the experimental groups. The Y-maze analysis represented (**K**) total distance covered (cm) and (**L**) spontaneous alteration behaviors. (**M**,**N**) indicating MWM and y-maze trajectories. For the behavioral study, the number of mice (*n* = 16/experimental group) was used. Asterisk sign (*) indicated significant difference from the normal saline-treated (Cont.) group; hash sign (#) indicated significant difference from LPS-alone treated group. Significance: * # = *p* ≤ 0.05 and **= *p* ≤ 0.01. n.s = non significance.

Early studies have indicated that the aberrant activation of JNK potentially contributes to synaptic loss and cognitive deficits/memory impairment in rodents [38]. Importantly, others also reported that systemic administration of LPS-injection-induced synaptic dysfunction and memory impairment [7]. In order to analyze the effects of V.A on LPS/JNK-mediated synaptic loss, we performed western blotting, immunofluorescence, and behavioral analysis. Our immunoblot results showed that compared to saline-treatment, systemic administration of LPS-injection significantly decreased protein expression level of memory-related presynaptic proteins, including a postsynaptic density protein (PSD95) and synaptophysin (Syp) in cortex and hippocampus regions in the LPS-alone treated group. Interestingly, V.A + LPS co-treatment significantly increased protein expression levels of synaptic markers (PSD95 and Syp) in comparison with the LPS-alone treated group (Figure 5A–C). To further ascertain these results, we used confocal microscopy. In accordance with our immunoblot results, our immunofluorescence analysis also suggested that PSD95 immunoreactivity was significantly decreased (cortex and DG region of hippocampus) in LPS-alone treated group in comparisons with normal saline-treated (control) mice. Interestingly, V.A + LPS co-treatment significantly increased immunofluorescence reactivity of PSD95 in both the indicated regions in comparison with the LPS-alone treated group (Figure 5D,E).

Likewise, synaptic dysfunction, it has also been demonstrated that systemic administration of LPS-injection leads to memory/cognitive impairment [6]. Therefore, to analyze the effect V.A on mouse behavior and memory, we therefore, performed behavior analysis, including Morris water maze (MWM) and Y-maze tests. For this purpose, we initially trained all experimental mice in an MWM task where they were challenged to reach a hidden platform that had been submerged and then measured the time needed to reach the hidden platform. In the MWM test, mean latency (time in seconds required to find hidden platform) gradually reduced in all experimental mouse groups over training days, except for the LPS-alone treated group, which showed longer latency than the normal saline-treated (control) mice group, indicating impaired spatial learning and memory abilities (Figure 5F,G). However, compared with LPS-alone treated group, this effect has been reversed by V.A + LPS co-treatment that significantly improved memory performance, as shown by the mice taking less time to reach the hidden platform. Next, we performed the probe test by removing the hidden platform. We found that the time spent in the target quadrant (Probe test: Figure 5H) and the number of platform crossings (Probe test: Figure 5I) was increased significantly in the V.A + LPS-co-treatment group compared with the LPS-alone treated group, showing that V.A reduced LPS-induced memory impairment. In addition, we found no significant difference in swimming speed (cm/s) among all the experimental mice implying that normal physiological functions of motor neurons and comparable ability to reach the hidden platform. Following the MWM test, next, we performed the Y-maze test to evaluate spatial working memory based on the percentage (%) of spontaneous alteration behavior. A higher percentage (%) of spontaneous alteration behavior was an indication of increased cognitive performance. We found that the LPS-alone treated group covered less distance (cm) and exhibited a substantially lower percentage (%) of spontaneous alterations than the normal saline-treated (control) mice group, suggesting impaired working memory. However, compared to the LPS-alone treated group, the V.A + LPS-co-treatment mice showed a marked increase in spontaneous alteration behavior (%), suggesting that V.A mitigated short-term memory deficits in the LPS-alone treated group (Figure 5L).

**Figure 6:**
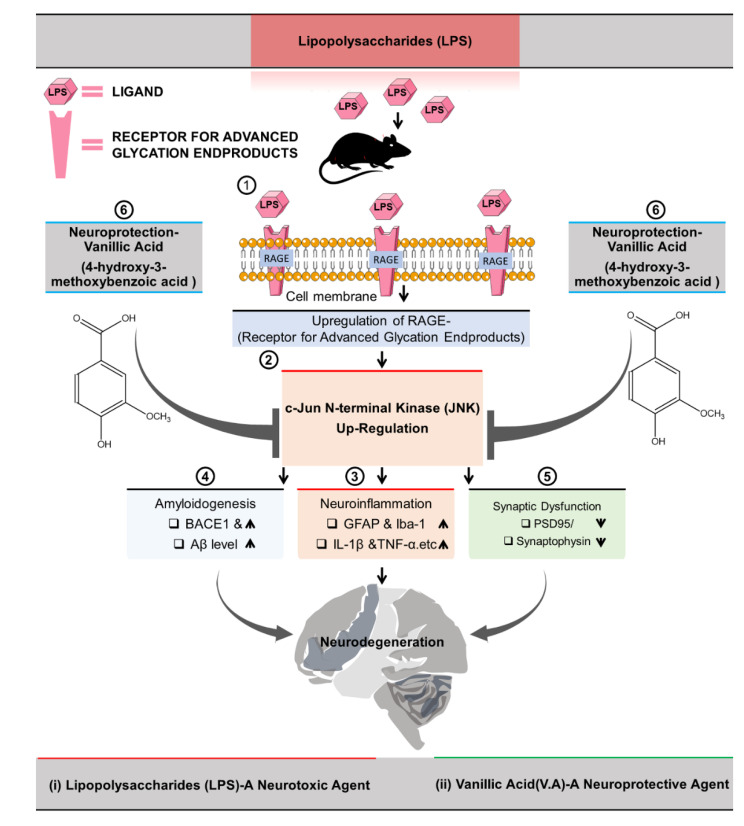
Suggested graphical representation of neuroprotective mechanism of vanillic acid against LPS-induced neurotoxicity in mice brain. ① LPS (intraperitoneal injections (I.P.) 250 μg/kg/day) via RAGE result in neurodegeneration. ② in this process, LPS-activated p-JNK has a central role in activating various downstream signaling cascades lea ding to ③ neuroinflammation: Resulting from increased in expression level of p-NF-κB, TNFα, IL-1β, and COX-2. ④ amyloidogenesis: Resulting from increased in expression level BACE-1 and Aβ. ⑤ Synaptic dysfunction: Resulting from reduction in the expression level of synaptic makers (PSD-95, SYP). ⑥ vanillic acid (I.P. 30 mg/kg) reduced LPS-induced neurotoxicity might via inhibition of LPS/RAGE-mediated elevated c-Jun N-terminal kinase in mice brain.

Taken together, these findings suggested that V.A has synaptoprotective and memory-enhancing effects that significantly reversed LPS-induced synaptic dysfunction and memory/cognitive impairment (Figure 6).

## 3. Discussion

Recently, there has been a growing awareness of the importance of natural sources (plant phenolic) in research studies to combat neurological disorders [24]. V.A abundantly occurs in edible plants and fruits with numerous pharmacological activities including anti-hypertensive, antioxidant, anti-inflammatory, and neuroprotective effects [25,27,28,31]. In this study, we investigated the neuroprotective potential of V.A against LPS-induced neurotoxicity. Taken together, we suggested that V.A inhibits LPS-induced neuroinflammation, amyloidogenesis, synaptic dysfunction, and memory impairments might via inhibition of RAGE/JNK-signaling pathway in mouse brain (Figure 6).

Persistent chronic neuroinflammation is a pathological mechanism that develops into several neurodegenerative diseases [6]. Increasing evidence has indicated that RAGE is a key player in regulating inflammation-driven neurodegeneration [40]. RAGE is ubiquitously expressed on immune/inflammatory cells, including microglia and astrocytes [12]. LPS, an endotoxin from the bacteria’s outer membrane, is widely recognized as a potent inflammatory agent [41]. Importantly, in addition to the LPS binding to toll-like receptor 4(TLR-4) in the induction of activated gliosis, evidence also confirmed the physical binding/interaction of LPS with RAGE [6,42,43]. This ligand/receptor interaction induces the RAGE activation and therefore enhances brain’s neuroinflammation [44,45]. Both receptors share similar signals for inflammation and have been involved in the pathogenesis of many diseases [46]. Notably, many studies have shown that LPS increases the expression level of RAGE proteins [15,34]. On the other hand, studies have reported that plant-derived compounds (polyphenol) significantly down-regulated RAGE level and therefore, have potential beneficial roles/therapeutic efficacy in the treatment of various diseases [47,48]. Likewise, herein, we also examined the therapeutic effect of V.A. Consistently with the previous reports, our results also showed that systemic LPS-injection significantly increased the protein expression level of RAGE in both regions (cortex and hippocampus) of mice brain. Interestingly, V.A + LPS co-treatment significantly reduced LPS-induced increase in the protein expression level of RAGE. Additionally, LPS-induced elevated RAGE expression (found in various brain cells), glial cells, in particular, are considered important brain residential macrophages which play significant role not only in neuronal function, neurogenesis, and regeneration but also as an initial line of immune defense in response to any form of brain injury due to its capability to phagocytose the toxic product and trigger cytotoxic factor [49]. In CNS, glial cells (microglia and astrocytes) are significant neuroinflammatory modulators that respond immediately to complaints such as infectious agents and injuries [6]. In chronic neuroinflammation, activated microglia and astrocytes disrupt homeostasis, and therefore are involved in all inflammatory CNS diseases [6,50]. Importantly, previous studies have proven that the peripheral/systemic application of LPS induces activation of both the astrocyte and microglia within the brain of mice [41,51]. On the other hand, numerous studies have shown that natural phenolic compounds halted gliosis triggered by LPS [6,36]. Likewise herein, in accordance with previous studies, we also found that V.A + LPS co-treatment inhibits LPS-induced activated gliosis by reducing the expression level of GFAP (astrocytes) and Iba-1 (microglia) in the mouse brain. Based on these findings, we suggest that the V.A is effective in reversing LPS-mediated elevated expression of RAGE and glial cells in the mouse brain.

Studies have suggested that activation of RAGE via its ligands (ligand-RAGE interaction) triggered a complex cascade of signaling pathways events, including MAPK signaling cascade [17,52]. Of importance, many studies reported that RAGE triggered JNK phosphorylation [45,52,53]. The JNK protein; is a mitogen-activated protein kinase required for the induction of NF-κB activation [17]. It has been known that a strong association exists between activated JNK and NF-κB pathways [54]. The NF-kB transcription factor is among the major pro-inflammatory gene transcription modulators triggered by LPS via TLR4 and RAGE [55]. The activation and nuclear translocation of the NF-kB leads to overproduction and release [6] of several pro-neuroinflammatory mediators as documented in neuroinflammatory neurodegeneration [4]. Notably, a, previous, considerable number of studies have also shown that systemic LPS administration dramatically increases the protein expression level of p-JNK, p-NF-κB, and its associated inflammatory mediators including TNF-α, COX-2, IL-1β, and nitric oxide synthase 2 (NOS-2) in cortex and hippocampus regions in rodent [6,36]. On the other hand, studies have reported that plants-derived compounds (polyphenol) significantly down-regulated elevated p-JNK, p-NF-κB, and its associated inflammatory mediators and therefore; have potential beneficial roles/therapeutic efficacy in treatment of various diseases [7,56]. Likewise, in the current study, our findings also supported the previous findings and elucidated that V.A + LPS co-treatment suppressed p-JNK, p-NF-κB, and its associated pro-inflammatory mediators (TNF-α, IL-1β, and NOS-2) in the indicated region (cortex and hippocampus) of mouse brain. Based on these findings, we suggest that the anti-inflammatory effect of V.A against LPS-induced neuroinflammation might be possibly via inhibition of JNK-mediated p-NF-κB signaling pathway (Figure 6).

Emerging research studies have indicated that neuroinflammation and RAGE’s involvement in several signal pathways contribute to the onset of AD pathogenesis, underlying the development of Aβ (amyloid plaque formation) and aberrant tau hyperphosphorylation (NFTs formation) [49,57]. Particularly, RAGE-mediated JNK phosphorylation [45,53] has been reported in the processing of amyloid-βeta precursor protein (AβPP), Aβ production/accumulation, and tau protein hyperphosphorylation in AD brains [20,58]. Other studies also supported that JNK is the main kinase for the phosphorylation and cleavage of amyloid precursor protein (APP) resulting in the activation of the amyloidogenic process of the protein. [59,60,61]. Patients with AD have been reported with increased expression of phosphorylated JNK (p-JNK) [59,62]. Notably, previous studies have shown that systemic administration of LPS-injection significantly elevated the protein expression levels of p-JNK level, AD markers (APP, BACE-1, Aβ, and p-Tau) that ultimately developed into axonal pathology and dendritic degeneration in rodent brains [14]. On the other hand, JNK inhibition with plant-derived compounds (polyphenol) or natural inhibitors has anti-amyloid functions (drastic decrease in Aβ_1–42_ peptide levels) that halts AD progression [59,63,64]. Likewise, in agreement with the previous findings, in the current study, our results also indicated that V.A + LPS co-treatment significantly reversed LPS-induced increases in BACE-1 and Aβ levels in the indicated region (cortex and hippocampus) of mouse brain, indicating that anti-AD-like effects of V.A. Based on these findings, we suggest that the anti-amyloidogenic effect of V.A against LPS-induced amyloidogenesis might be possible via inhibition of the JNK-mediated amyloidogenic signaling pathway.

It has been reported that synaptic proteins play an important role in synaptic plasticity and memory [65,66]. An altered synaptic (pre- and postsynaptic) proteins are strongly connected with memory impairment and cognitive dysfunctions [6]. In this aspect, studies have shown the imported role of JNK in synaptopathy/synaptic degeneration and memory dysfunction in experimental animal models [22,67,68]. Notably, a growing body of evidence has also shown that LPS-induced synaptic dysfunction by decreasing protein expression level of memory-associated proteins (a postsynaptic protein; PSD-95, presynaptic proteins; synaptophysin (SYP); synaptosomal associated protein (SNAP-25), and syntaxin-1) [6] and memory impairment [69,70,71]. On the other hand, plant-derived compounds (polyphenol) have been well studied due to their ability to improve synaptic functionality (increased expression level of deregulated synaptic markers) and learning/memory performance/functions in rodent animal models [72,73,74,75]. Likewise, in accordance with previous findings, herein our results also indicated that systemic administration of LPS-injection results in synaptic dysfunction by reducing protein expression level of PSD-95 and SYP in both cortex and hippocampus regions of mouse brain. Interestingly, V.A + LPS co-treatment significantly restored the expression of these synaptic protein markers (PSD95 and Syp) to basal levels (Figure 5) and therefore, alleviates LPS-induced synaptic impairment. Similarly, our behavior analysis (MWM and Y-maze tests) also indicated that systemic administration of LPS-injection significantly impaired cognitive and learning behavior. However, V.A + LPS co-treatment substantially reversed these effects and improved cognition, spatial learning, and memory processing (Figure 5). Taken together, these findings demonstrated that V.A is effective in reversing LPS-induced synaptic dysfunction and memory impairment in mouse brains.

## 4. Materials and Methods

### 4.1. Chemicals

LPS and V.A were purchased from Sigma Aldrich Chemicals Company (St. Louis, MO, USA). Dimethyl sulfoxide (DMSO) was purchased from Promega (Madison, WI, USA).

### 4.2. Mouse Strain, Acclimatization/Housing and Ethical Aspects

Wild-type males (C57BL/6N mice, 8 weeks old, body weight: 25–30 g) were purchased from Samtako Bio (Osan, South Korea). The mice were acclimatized under a 12-h/12-h light/dark cycle at 23 °C at 60 ± 10 percent humidity and provided with food and water ad libitum for 1 week in the university animal house. The mice were maintained and treated in accordance with the guidelines of the Institutional Animal Care and Use Committee (IACUC) approved by the Applied Life Science Division, Gyeongsang National University, South Korea. Every effort has been made to reduce the animals’ suffering. All methods and experimental procedures were carried out with mice in accordance with compliance with the decided to adopt guidelines (1 July 2020. Approval ID: 125). The IACUC Division of Applied Life Science, Gyeongsang National University, South Korea, has approved all experimental protocols.

### 4.3. Study Designing, Experimental Animals Grouping, and Their Drug Treatment

**Figure 7:**
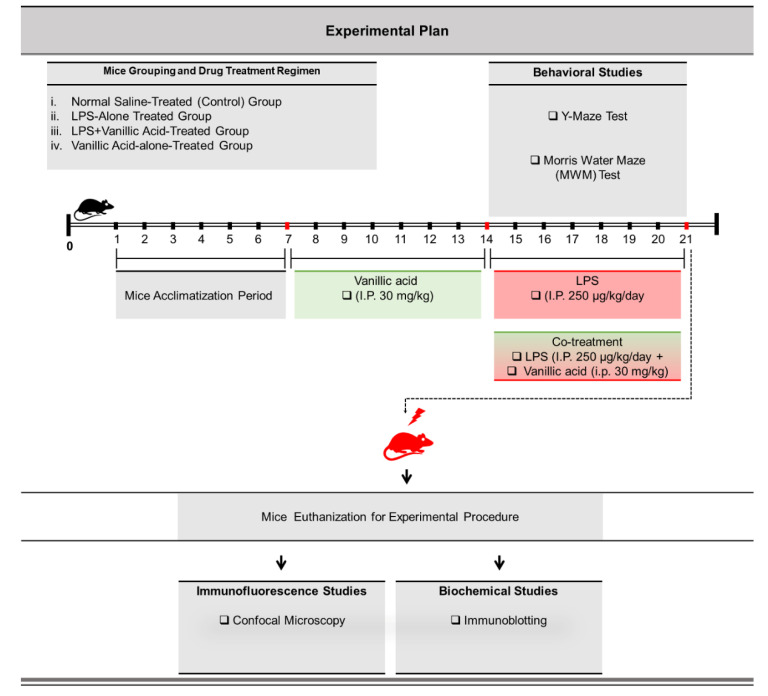
Schematic design of the current research study, classification of animals into the experimental grouping, drug dosage treatment, behavioral analyses, biochemical and morphological experimental approach for the entire research study. The experimental mice were randomly divided into four groups. (i) mice treated with normal-saline as a vehicle (two weeks; I.P.); normal saline-treated (Cont.) group. (ii) mice treated with vehicle (one week) and LPS (I.P.: 0.25 mg/kg/day; one week); LPS-alone treated group. (iii) mice treated with LPS (0.25 mg/kg/day; one week) and V.A (I.P.: 30 mg/kg/day; two weeks); i.e., one week before the LPS treatment and one week co-treated with LPS); V.A + LPS co-treatment group. (iv) mice treated with V.A (I.P.: 30 mg/kg/day; two weeks); V.A-alone treatment group. After completion of behavioral analyses, experimental mice were euthanized and further subjected to immunoblotting and immunofluorescence analyses.

To accomplish our hypothesis, the following studies (Figure 7) were designed as per our previous reports [24]. After acclimatization was complete, the experimental mice were divided into the following four categories (*n* = 16 mice/group).

1. Normal saline-treated (control) group: Mice treated with normal saline (i.p. 0.9% saline for 14 days).
2. Lipopolysaccharide-alone treated group: Mice treated with LPS (i.p. 250 μg/kg/day, for 7 days).
3. LPS + vanillic acid-co-treated group: Mice treated with LPS (i.p. 250 μg/kg/day) for 7 days and V.A (i.p. 30 mg/kg for 14 days (7 days prior to the LPS and 7 days co-treated with LPS).
4. V.A-alone treated group: Mice treated with V.A (i.p. 30 mg/kg for 14 days alone (V.A).

### 4.4. Behavioral Tests

After administration and completion of drug treatment, we performed behavioral studies, including the Morris water maze and Y-maze test.

The MWM test is the most well-known and well-established task for analyzing memory functions, so we did the MWM as described earlier [18]. The experimental setup (apparatus) consists of a circular water tank (100 cm in diameter; 40 cm in height) filled with water (23 ± 1 °C) to a depth of 15.5 cm, which was made opaque by adding white ink. In the center of one quadrant, a transparent escape platform (diameter 4.5 cm, height 14.5 cm) was hidden (1 cm) below the surface of the water. To find this hidden platform, all experimental mice were initially trained for 5 consecutive days with 4 trial sessions (120 s/trial) per day to test the reference memory. Each mouse’s escape latency (latency to find the hidden platform underwater) was calculated in each trial. On next day, final escape latency and probe tests were performed in order to evaluate memory consolidation. In the probe test, each mouse was allowed to swim freely for 60 s after removing the platform. In the probe trial, the time spent in the target quadrant (where the platform was located during hidden platform training), the time spent in the other three quadrants (left, right, and opposite), number of platform crossings and swimming speed was measured. All data were recorded using Visual/Video Tracking Software (SMART, Panlab Harvard Apparatus; Bioscience Company, Holliston, MA, USA).

Next, for the Y-maze task, a Y-maze apparatus was used (20 cm in height, 50 cm in length, and 10 cm in width underneath) [76]. The apparatus was built of black-painted wood and used to assess spatial working memory. In brief, individual mice were placed in the center of the maze and allowed to move freely (3- to 8-min sessions) over different time intervals. The sequence of entries to the arm was recorded digitally. In addition to total distance covered (cm), the spontaneous alteration percentage (%) was calculated as defined as [successive triplet sets (consecutive entries into three different arms)/total number of arms entries−2× 100. A higher percentage (%) of spontaneous alteration behavior reflected improved memory and cognitive function.

### 4.5. Protein Extraction for Biochemical Analysis from Mice Brains

After completion of behavioral studies, For biochemical/western blotting, the experimental mice/animals (*n* = 8) were brought into the surgical room and anesthetized with Rompun (Xylazine; 0.05 mL/100 g body weight) and Zoletil (ketamine; 0.1 mL/100 g body weight) as described previously [6]. Once these mice were anesthetized, they were then euthanized by decapitation. The brain tissue (cortex and hippocampus) was carefully removed and stored at −80 °C. These brain tissues were homogenized in PRO-PREP^TM^ protein extraction solution as per the instructions of the manufacturer (iNtRON Biotechnology, Burlington, NJ, USA). These samples were then centrifuged for 25 min at 4 °C at 13,000 rpm. The supernatants were thus collected and kept at −80 °C before reused for further processing.

### 4.6. Western Blot Analysis

Western blotting was performed as reported earlier [6,77]. Briefly, the concentrations of protein were measured in the samples (BioRad protein assay kit, BioRad Laboratories, Hercules, CA, United States). Equal protein amounts (15–30 μg) were electrophoresed on 12–15% SDS-PAGE gel before transferred to a membrane of polyvinylidene difluoride (PVDF). In parallel, a protein marker (GangNam-STAIN, iNtRON Biotechnology) was run to detect the molecular weights of proteins. The membranes were blocked using 5 percent skim milk to reduce the non-specific binding membrane and incubated with primary antibodies overnight at 1:1000 dilution at 4 °C (Table 1). Chemiluminescence reagent (Amersham ECL Advance Western Blotting Detection Reagent) has been used to detect the immune reaction. The X-ray films were scanned, and the bands’ optical densities were measured via Sigma Gel computer-based software version 1.0 (SPSS, Chicago, IL, USA).

### 4.7. Brain Tissue Collection and Sample Preparation for the Immunofluorescence Staining

After completion of behavioral studies, For morphological/immunofluorescence analysis, the experimental mice/animals (*n* = 8) were brought into the surgical room and anesthetized with Rompun (Xylazine; 0.05 mL/100 g body weight) and Zoletil (ketamine; 0.1 mL/100 g body weight) as described previously [6]. These mice were perfused transcardially with normal saline solution (0.9%) followed by paraformaldehyde (4%). The mice brain tissue was removed immediately and fixed at 4 °C for 72 h with ice-cold paraformaldehyde. After this, they were submerged for 72 h in sucrose phosphate buffer (20%). All mice’s brains were frozen in optimum cutting temperature (O.C.T) compound (tissue-Tek O.C.T compound medium, Sakura Finetek USA, Inc., Torrance, CA, USA) and further cut into coronal sections of 14 μm using a CM3050C cryostat (Leica, Nussloch, Germany).

### 4.8. Immunofluorescence Staining

The immunofluorescence staining was proceeded with few modifications as mentioned previously [6,77]. In brief, each slide covering brain sections was washed two times in 0.01 M PBS (phosphate-buffered saline;10 min), followed by adding and incubating with 1X proteinase K for 5 min at room temperature. Subsequently, each slide was washed twice for an additional 5 min before incubation in blocking solution (1 h) containing normal serum (2%) and Triton X-100 (0.3%) in PBS (0.01 M) according to the treatment of antibody. After blocking the slides containing brain tissue, they were incubated with primary antibodies (1:100 ratio in 1% PBS, i.e., 0.01 M) overnight at 4 C (Table 1) followed secondary antibodies; tetramethylrhodamine isothiocyanate (TRITC)/fluorescein isothiocyanate (FITC)-labelled antibodies for 2 h (1:50 dilution in 1% 0.01 M PBS; Santa Cruz Biotechnology, Dallas, TX, United States). Tissue slides were washed two times for five minutes after they had been incubated with a secondary antibody. Then, 4′, 6′-diamidino-2-phenylindole (DAPI) was used before the slides were mounted with mounting media (Dako Fluorescence Mounting Medium, REF S3023, Carpinteria, CA, USA) were mounted on slides containing brain tissue. Finally, the slides section was examined through a confocal laser-scanning microscope (Flouview FV 1000, Olympus, Tokyo, Japan).

### 4.9. Antibodies

All antibodies (primary and secondary) used in the present study are provided in the table shown below (Table 1).

### 4.10. Data and Statistical Analyses

In brief, ImageJ software (version 1.50, NIH, https://imagej.nih.gov/ij/, USA) has been used to analyze all scanned immunoblots data (density in A.U and morphological data (integrated density in A.U), respectively. GraphPad Prism 6 (San Diego, CA, USA) software was used to generate histograms/graphs. For comparisons of differences among the experimental groups, statistical analyses were performed using one-way analysis of variance (ANOVA) followed by Tukey’s multiple comparison test. The data expressed are presented as the means ± standard error of mean (SEM) and are representative of the three independent experiments. Differences between groups were considered statistically significant at *p* < 0.05 (significance: * # *p* ≤ 0.05; ** ## *p* ≤ 0.01; and *** ### *p* ≤ 0.001). An asterisk (*) indicates a significant difference from the control saline-injected treated group, hashtag (#) indicates a significant difference from the LPS-injected/treated groups, while the phi sign (Φ) indicated no significance from the normal saline-treated control group. (All western blots and histogram are represented as Cont. = normal saline-treated (control) mice, LPS = LPS-alone treated group, V.A + LPS = LPS and V.A co-treated group, and V.A = V.A alone-treated group).

## 5. Conclusions

In summary, the findings of the current study demonstrated that V.A is a potential neurotherapeutic candidate that markedly reversed systemically-injected LPS-induced glial cells activation, neuroinflammation, amyloidogenic makers, and synaptic dysfunctions/memory impairment in mouse brains. We propose that these underlying potent neuroprotective effects of V.A against LPS-induced neurotoxicity might be due to inhibition of LPS/RAGE interaction mediated JNK-pathway as described in the schematic diagram (Figure 7). Based on these findings, we suggest that V.A is a safe, effective, and a promising neurotherapeutic agent. However, more future studies are highly encouraged to further evaluate the underlying molecular mechanism and role of V.A in neuroinflammatory and various age-related neurodegenerative disorders, particularly in AD.

## Figures and Tables

**Table 1:** A detailed list of antibodies and their information used for western blot and immunofluorescence.

Antibody	Host	Catalog	Application	Dilution	Manufacturer
Anti-β-Actin Antibody	Mouse	sc-47778	WB	1:1000	Santa Cruz Biotechnology (Dallas, TX, USA)
Anti-p-JNK Antibody	Mouse	sc-6254	WB and IF	1:1000/1:100	Santa Cruz Biotechnology (Dallas, TX, USA)
Anti-PSD-95 Antibody	Mouse	sc-71933	WB and IF	1:1000/1:100	Santa Cruz Biotechnology (Dallas, TX, USA)
Anti-Synaptophysin	Rabbit	sc-7568	WB	1:1000	Santa Cruz Biotechnology (Dallas, TX, USA)
Anti-BACE-1 Antibody	Mouse	sc-33711	WB	1:1000	Santa Cruz Biotechnology (Dallas, TX, USA)
Anti-Aβ Antibody	Mouse	sc-28365	WB and IF	1:1000/1:100	Santa Cruz Biotechnology (Dallas, TX, USA)
Anti-Iba-1 Antibody	Mouse	sc-32725	WB	1:1000	Santa Cruz Biotechnology (Dallas, TX, USA)
Anti-GFAP Antibody	Mouse	sc-33673	WB and IF	1:1000/1:100	Santa Cruz Biotechnology (Dallas, TX, USA)
Anti-IL-1βeta Antibody	Mouse	sc-7884	WB and IF	1:1000/1:100	Santa Cruz Biotechnology (Dallas, TX, USA)
Anti-COX-2 Antibody	Rabbit	SC: 7951	WB	1:1000	Santa Cruz Biotechnology (Dallas, TX, USA)
Anti-TNF-α Antibody	Mouse	sc-52746	WB	1:1000	Santa Cruz Biotechnology (Dallas, TX, USA)
Anti-RAGE Antibody	Mouse	sc-80652	WB and IF	1:1000/1:100	Santa Cruz Biotechnology (Dallas, TX, USA)
Anti-p-NF-Κb Antibody	Mouse/Rabbit	SC 8008	WB and IF	1:1000/1:100	Santa Cruz Biotechnology (Dallas, TX, USA)

(WB: Western blotting and IF: Immunofluorescence).

## Data Availability

The authors hereby declares that the data presented in this study will be presented upon request from the corresponding author.

## Abbreviations

LPS	Lipopolysaccharide
V.A	Vanillic acid
CNS	Central nervous system
p-JNK	c-Jun n-terminal kinase
NF-KB	nuclear factor kappa-light-chain-enhancer of activated B cells
TNF-α	tumor necrosis factor alpha
IL1-β	interleukin-1 β
COX-2	cyclooxygenase
Aβ	amyloid-β
BACE1	β-site amyloid precursor protein (APP)–cleaving enzyme 1
PSD-95	Post-synaptic density protein 95
